# Cryptochrome interaction networks across different tissues in *Drosophila melanogaster*

**DOI:** 10.1186/s13062-025-00696-x

**Published:** 2025-11-28

**Authors:** Milena Damulewicz, Francesco Gregoris, Davide Colaianni, Filippo Cendron, Alberto Biscontin, Giovanni Minervini, Gabriella M. Mazzotta

**Affiliations:** 1 Department of Cell Biology and Imaging, Institute of Zoology and Biomedical Research, Faculty of Biology, Jagiellonian University, Kraków, Poland; 2 Department of Biomedical Sciences, University of Padova, Padova, Italy; 3 Department of Biology, University of Padova, Padova, Italy; 4 Department of Agronomy, Food, Natural Resources, Animals and Environment, University of Padova, Padova, Italy; 5 Department of Agricultural, Food, Environmental and Animal Sciences, University of Udine, Udine, Italy

**Keywords:** *Drosophila*, CRYPTOCHROME, Protein-protein interaction, Networks, RNA regulation, SQUID

## Abstract

**Background:**

*Drosophila* CRYPTOCHROME (dCRY) is a blue light-sensitive protein involved in various biological processes, including photoreception, circadian rhythm regulation, synaptic plasticity, and magnetoreception. Its circadian function is strictly connected with light: upon light exposure, dCRY undergoes a conformational change, becoming active and binding to various proteins. However, it can also form complexes in the absence of light, with its function varying depending on the cell type in which it is expressed.

**Results:**

Here, we use an experimental approach based on co-immunoprecipitation followed by mass spectrometry analysis, obtaining the in vivo interactome of dCRY in two distinct cell populations - retina photoreceptors and glial cells - at two specific time points: just before lights-on (ZT0) and one hour after lights-on (ZT1). To gain deeper insights into the functional dynamics of dCRY, we constructed reliable protein-protein interaction networks in both cell types and across the two experimental conditions, revealing a complex landscape of interactions. Additionally, we explored the biological pathways associated with the identified dCRY interactors, highlighting several tissue- and time-specific enrichments. We focused on RNA-related pathways, indicating that dCRY and its interactors are involved in regulating RNA metabolism in photoreceptors at ZT0 and in glial cells at ZT1. Finally, as a case study, we further investigated the functions of the RNA-binding protein SQUID, found to interact with dCRY in both tissues. Notably, the impaired circadian locomotor behavior exhibited by *Squid* mutant flies accounts for the involvement of this hnRNP in the generation of the circadian rhythmicity.

**Conclusions:**

In conclusion, our work provides the first tissue- and time-specific dCRY interactome, offering valuable insights into previously unrecognized biological processes in which it may be involved. Specifically, its potential role in the regulation of RNA metabolism contributes crucial evidence concerning the relationship between the circadian clock and RNA metabolism, thereby laying the groundwork for future research in this area.

**Supplementary Information:**

The online version contains supplementary material available at 10.1186/s13062-025-00696-x.

## Background

*Drosophila* CRYPTOCHROME (dCRY) is a blue light photoreceptor that conveys photic signals to the circadian clock [1, 2]. Mutants show many abnormalities in light sensing: the hypomorphic *cry*^*b*^ mutants fail in the clock synchronization to light [3, 4], while flies overexpressing *cry* are supersensitive to photic stimuli [5, 6]. Structurally, dCRY is closely related to the DNA-repair (6 − 4) photolyase enzymes: it harbors an N-terminal photolyase homology region (PHR) and a C-terminus tail (CTT) [7, 8], responsible for the light-resetting of the circadian clock [2, 9, 10]. Indeed, upon light exposure, dCRY undergoes a conformational change [11, 12], which promotes the binding to TIMELESS [13, 14], and its degradation through the proteasome [15].

Besides its role in the light-dependent synchronization of the clock, dCRY is involved in many other aspects in the fly neurophysiology and behavior (reviewed in Damulewicz and Mazzotta, 2020 [16]): in the visual system, it can be a clock component able to repress the CLK/CYC mediated transcription of *per* and *tim* [17], it has a role in modulating the circadian visual sensitivity [18, 19] and synaptic plasticity [20, 21] and in optimizing vision under different light intensity regimes [22]. Furthermore, it is involved in the action potential modulation of neurons [23–25], and in several aspects of magnetoreception [26–30].

Many of these non-specifically photoreceptive functions of dCRY have been revealed thanks to the dissection of its macromolecular complexes. For example, the above-described roles in the visual system are mediated by its interaction, not always dependent on light, with the phototransduction pathway proteins and with CALMODULIN [18, 31], ACTIN [22], or the synaptic protein BRUCHPILOT [19].

Protein-protein interactions (PPIs) are crucial to most biological processes, and several efforts have been made to generate large-scale maps of protein interactomes in *Drosophila*, to build new hypotheses regarding protein networks and function [32–38].

dCRY harbors several protein-protein interaction domains and possesses linear motifs evolutionarily conserved in different species, mostly located in the CCT [10, 18], allowing the formation of multiprotein complexes that can vary in composition in different organs and tissues, and therefore account for the panoply of roles exhibited by this photoreceptor. This raises the interest in studies aimed at exploring dCRY interactome, even more considering the important effect that light and circadian timing have on its activity and stability.

Very recently, a comprehensive analysis of in vitro dCRY interactome in S2 cells has been published [39]. By using an innovative approach that allows overriding the technical difficulties due to the degradation of dCRY or its partners after the formation of (light-dependent) complexes, this piece of work uncovered several novel interactors of dCRY and, more generally, paved the way for future studies regarding light- and time-dependent protein interactions in *Drosophila* [39].

Here we present the results of a project that utilized a classical experimental approach, based on co-immunoprecipitation (Co-IP) followed by mass spectrometry (MS) analysis, to draw the in vivo interactome of dCRY in glia and photoreceptors at two specific time points, ZT0 (before lights-on) and ZT1 (after 1 h of light exposure), to expand our understanding of its functions in these neuronal populations in both light and darkness. In this context, we also compared our datasets with the aforementioned work by Ozcelik et al. [39], highlighting how both the “state of the art” (Co-IP/MS) and more innovative techniques are essential tools for discovering novel PPIs associated with dCRY. After constructing reliable PPIs networks in the two neuronal populations and across the two experimental conditions under study, we performed a gene ontology (GO) analysis on dCRY interactors to elucidate their functions and the biological pathways in which they were involved. Interestingly, using two different filters, we were able to highlight how many of the resulting pathways were characterized by a tissue- and/or time-specific enrichment. Of those, we focused our attention on the RNA-related terms, suggesting that dCRY and its interactors are involved in the regulation of RNA metabolism within photoreceptors at ZT0 and glia at ZT1. Finally, we demonstrated the involvement in the generation of the circadian rhythmicity of a heterogeneous ribonucleoprotein, SQUID, which was found to interact with dCRY in both tissues after light exposure.

## Methods

### Fly strains

The following strains of *Drosophila melanogaster* were used: CantonS (wild type), *w*^*1118*^, *repo*-Gal4 (BDSC #7415), GMR-Gal4 (BDSC #8440), *UAS-HAcry* [9], *yw; tim*-Gal4 [3], *w; P{wHy}sqd*^DG09709^ [40]. The latter is a mutant line generated by P-element insertion in the first intron of the gene [41] that highly reduces its expression [42]. Consistent with other *Squid* mutant alleles [43, 44], this mutant is not viable when the insertion is in homozygosity; therefore, it was maintained and used for the analyses as a balanced stock (hereinafter referred to as *Squid* mutant) over MKRS chromosome. Flies were maintained on a standard cornmeal medium under 12:12 light-dark (LD) regime and at constant 23 °C.

### Co-Immunoprecipitation and mass spectrometry analysis

#### Co-Immunoprecipitation

Proteins were extracted from 50 male fruit flies’ heads per sample. Head extracts from overexpressing HACRY (a hemagglutinin (HA)-tagged form of dCRY) flies (*repo* > HACRY and GMR > HACRY) and CantonS control flies, raised in 12:12 LD cycles and collected at the specified time points (ZT0 and ZT1, respectively), were subjected to co-immunoprecipitation in the number of 3 independent samples for each genotype at each time point. Details of the Co-IP protocol are reported in the Supplementary Materials. Immuno-complexes were then analyzed by Mass Spectrometry.

#### Protein identification by mass spectrometry

Mass Spectrometry analysis was performed at the Institute of Biochemistry and Biophysics, Polish Academy of Sciences. Details of the protocol are reported in the Supplementary Materials. Briefly, beads bounded proteins were subjected to digestion with trypsin and processed using single-pot solid-phase-enhanced sample preparation (SP3). Peptide mixture was analyzed using LC-MS system composed of Evosep One (Evosep Biosystems) coupled to an Orbitrap Exploris 480 mass spectrometer (Thermo Fisher Scientific) via Flex nanoESI ion source (Thermo Fisher Scientific). Samples were loaded onto disposable Evotips C18 trap columns (Evosep Biosystems) according to the manufacturer protocol with minor modifications. MS/MS data were pre-processed with the Mascot Distiller software (v. 2.4.2.0; Matrix Science), then obtained peptide masses and fragmentation spectra were matched to the UniProt database (Sprot: 569,516 sequences; 205,866,895 residues; Trembl: 249,308,459 sequences; 86,853,323,495 residues) with taxonomy filter *Drosophila melanogaster* (42,762 sequences) using the Mascot search engine (Mascot Daemon v. 2.4.0, Mascot Server v. 2.4.1, and Matrix Science). The list of identified proteins was exported to Excel MS software. The lists of dCRY interactors in the two neuronal populations at ZT0 and ZT1 obtained from CantonS control flies were used as a filter to minimize the inclusion of nonspecific proteins that are shared between the two neuronal populations for protein-protein interaction network (PPIN) composition.

### In Silico analysis

#### Network composition

Prey protein lists from glia and photoreceptors at two-time points were used to develop specific protein-protein interaction networks (PPINs) in Cytoscape 3.10.2 [45]. This software environment allows for directly managing and implementing biomolecular interaction networks. The initial step involves excluding prey proteins found in control samples (background) to focus on those specifically present in glia and photoreceptors. These curated protein lists were then used to retrieve interaction data using the STRINGapp in Cytoscape [46]. The app relies on the STRING database, from which we retained only experimentally validated interactions to generate PPINs for glia and photoreceptors at two time points (ZT0 = before lights-on, ZT1 = after 1 h of light exposure).

#### Scoring matrix for protein list comparisons

Ozcelik and colleagues [39] employed proximity-dependent biotinylation techniques, combining engineered BioID (TurboID) and APEX (APEX2) enzymes with mass spectrometry to identify in vitro dCRY interactors in *Drosophila* S2 cells. They categorized proteins as “significant” and “highly significant” based on the statistical reliability of protein binding. To compare the method used in this study and the new approach described in Ozcelik et al. (2024) [39], protein lists were generated using APEX and TurboID, with considerations for the effects of dark and light conditions. By intersecting our lists with the data from their paper, we developed a simple scoring system that explains the role of the proteins in the paper’s context and compares them to the experimental conditions applied in our study (Supplementary Table S1).

#### Enrichment analysis

The networks were enriched with Gene Ontology (GO) terms and Biological Pathways (WikiPathways) using the g: Profiler server [47]. Multiple testing correction was carried out with the g: SCS algorithm, the default method in g: Profiler, which is specifically designed for the hierarchical and non-independent structure of GO annotations. Traditional corrections such as Bonferroni or Benjamini–Hochberg FDR assume independent tests and can therefore be either overly conservative (Bonferroni) or suboptimal in this setting (FDR). In contrast, g:SCS provides an experiment-wide significance threshold equivalent to FDR control, but better tailored to the dependencies among GO terms. Results were considered significant at the standard threshold (q = 0.05), and a stricter cutoff (q = 0.001) was also applied to highlight only the most strongly enriched terms. Enrichment terms from all conditions were analyzed and visualized using the EnrichmentMap Cytoscape App pipeline [48], facilitating comparative analysis across different experimental conditions. These enrichment results were then integrated into the PPINs, connecting enriched terms with their corresponding proteins to facilitate biological interpretation.

### Western blot

Proteins were extracted from 50 male fruit flies’ heads per sample, and a total of 3 independent samples were analyzed for each genotype (*w*^*1118*^, *tim*-GAL4, and *tim* > HACRY) in the dark (ZT0) and in the light (ZT0 + 15 min light pulse). The *w*^*1118*^ strain was used as a positive control for the anti-SQUID antibody, while the parental line of the tested flies, *tim*-GAL4, was used as a negative control. Co-immunoprecipitation was performed as previously described [18]. Immunocomplexes were analyzed using mouse anti-SQUID (1:1000; DSHB #1B11), anti-HA (1:5000; Sigma Aldrich) as primary antibodies and anti-mouse IgG-HRP (1:5000; Sigma Aldrich) as secondary antibody.

### Immunocytochemistry

Flies raised at 23 °C in 12:12 LD were collected at ZT0 and fixed for 2 h at room temperature in 4% paraformaldehyde (PFA) in PBS and subsequently washed in PBS 3 times for 10 min at room temperature on a rotating wheel. Brains dissected in PBS were fixed in 4% PFA for 50 min at room temperature and permeabilized with 1% Triton X-100 (Sigma-Aldrich) for 10 min at room temperature. To avoid non-specific staining, a blocking step was set up by incubation in 1% BSA/0.1% Triton X-100 at room temperature for 2 h. Brains were then incubated with mouse anti-PDF (1:2500; DSHB #C7) diluted in 1% BSA and 0.3% Triton X-100 for 72 h at 4 °C and subsequently washed 5 times at room temperature on a shaker for 10 min with PBS. The blocking step was performed again in 1% BSA/0.1% Triton X-100 at room temperature for 1 h, and then the brains were incubated with the secondary antibody (goat anti-mouse Alexa Fluor 488, 1:250; Invitrogen) diluted in 1% BSA and 0.3% Triton X-100 for 1 h at room temperature. After 6 washes of 10 min with PBS at room temperature, the brains were mounted on a slide with Vectashield Antifade Mounting Medium (Vector Laboratories). The samples were stored at 4 °C until visualization through confocal microscope Leica TCS SP5. Several scans were performed for each brain at different depth to form a Z-series. The size of each section was approximately 1 μm.

### Behavioral analysis

Locomotor activity of individual flies was recorded for 3 days in LD and 7 days in DD using the *Drosophila* Activity Monitoring System (TriKinetics). Data were collected every 5 min and then analyzed in 30 min bins using spectral analysis and autocorrelation [49]. Under physiological conditions, adult *Drosophila* exhibit robust locomotor activity in anticipation of both lights-on and lights-off transitions, corresponding to the morning and evening onset of activity, respectively [50]. Morning anticipation was detected fly-by-fly examining the activity mean of 3 days under LD conditions and the Morning Index was calculated as in Seluzicki et al. (2014) [51]. Given the *Squid* mutant genetic background, it was compared with the *w*^*1118*^ strain, and a total of 96 (*Squid*) and 90 (*w*^*1118*^) flies were tested to generate sufficient statistical power. Statistical tests and significance are described in each figure caption.

## Results

### *Drosophila* CRY interactome networks in photoreceptors and glia

**Fig. 1:**
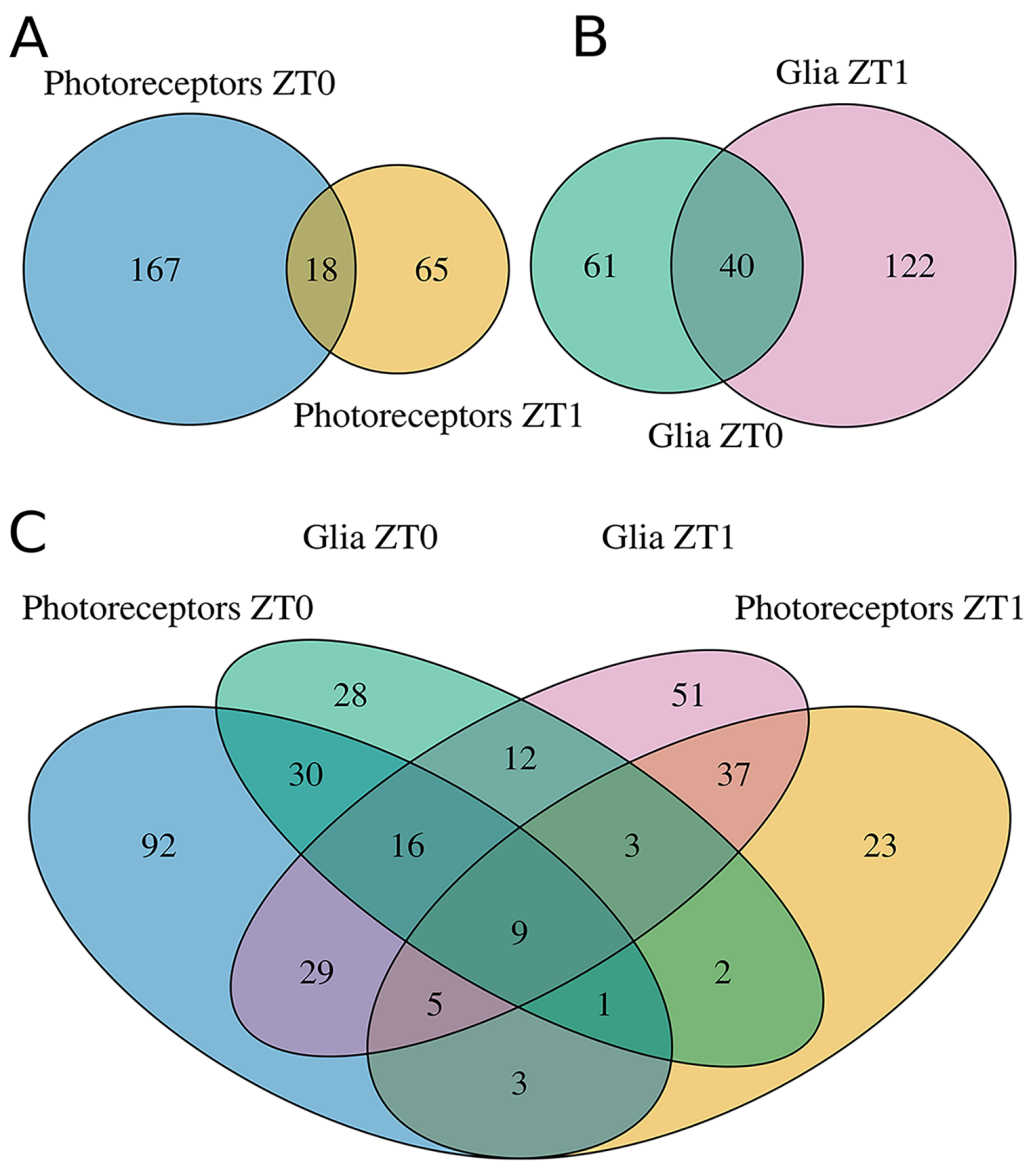
Venn diagrams summarizing dCRY interactors in the protein-protein interaction networks. The protein lists were used as input in Cytoscape’s STRINGapp to compose the network. Only experimentally validated interactions were retained, and the unconnected proteins were excluded. (**A**) Interactors distribution in the photoreceptors-based network at ZT0 and ZT1. (**B**) Interactors distribution in the glia-based network at ZT0 and ZT1. (**C**) Comparison between the two tissues (photoreceptors, glia) as well as the time periods (ZT0, ZT1)

To develop new hypotheses regarding dCRY functions, we opted to employ a proteomic strategy aimed at characterizing its interactome within two distinct neuronal populations: photoreceptors and glial cells. To do so, a co-immunoprecipitation assay, followed by mass spectrometry analysis, was performed on transgenic flies overexpressing a hemagglutinin (HA)-tagged form of dCRY (HACRY) specifically in the photoreceptors (by GMR-Gal4 driver) or in the glia (by *repo*-Gal4 driver). Flies were raised in 12:12 light-dark (LD) cycles and collected at *Zeitgeber* Time 24/0 (ZT0), before lights-on, and after one hour of light (ZT1). The lists of dCRY interactors specific for the two neuronal populations at two time points (Supplementary Tables S2 and S3) were used to construct specific protein-protein interaction networks (PPINs) in Cytoscape 3.10.2, aiming to directly identify proteins forming complexes with dCRY in a tissue- and/or time-specific manner. Our network exclusively includes experimentally validated interaction data obtained from the STRING database. The network composition is summarized in Fig. 1 in the form of Venn diagrams representing the number of shared interactors between the Photoreceptors dataset at ZT0 and ZT1 (Fig. 1A), the Glia dataset at ZT0 and ZT1 (Fig. 1B), and both datasets at both time points (Fig. 1C).

To validate the “state of the art” techniques (Co-IP/MS) employed in our study in comparison to the cutting-edge approach used in Ozcelik et al. (2024) [39], we applied a scoring matrix to the protein lists they obtained (see Methods section). With this comparison, we aimed to highlight how the previously described method was capable of effectively identify differentiated interactors based on ZT0 or ZT1. The results, shown in Supplementary Figures S1A, S1B and S2A, S2B (the complete scoring matrix is available in the Supplementary Tables S2 and S3), are summarized in Table 1. In brief, in the Photoreceptors dataset, we validated 36 proteins at ZT0 (55.56% of which were “significant”, and 30% were “highly significant”) and 25 proteins at ZT1 (44% of which were “significant”, and 27.27% were “highly significant”) among the dCRY interactors; on the other hand, in the Glia dataset, we validated 24 and 39 proteins at ZT0 (50% “significant”, 25% “highly significant”) and ZT1 (41.03% “significant”, 31.25% “highly significant”), respectively. Overall, we were able to validate 20–30% of the results obtained through innovative techniques employed by Ozcelik and colleagues [39], therefore demonstrating the robustness of our methodology, which allows us to identify phase-specific interactors while considering experimental parameters such as the use of validated techniques, ease of reproducibility, and cost containment.

**Table 1:** Summary of dCRY interactor proteins through different techniques

	Proteins mentioned in Ozcelik et al.	Significant proteins	Highly significant proteins
Photoreceptors ZT0 (185)	36/185	20/36	6/20
Photoreceptors ZT1 (83)	25/83	11/25	3/11
Glia ZT0 (101)	24/101	12/24	3/12
Glia ZT1 (162)	39/162	16/39	5/16

Here we reported the number of proteins in the protein-protein interaction networks (PPINs) produced from Co-IP/MS compared with those retrieved by Ozcelik and colleagues [39], who defined the interactors with relevant statistical values such as “significant proteins” and “highly significant proteins” based on p-values (see Methods section)

### Gene ontology analysis of *Drosophila* CRY interactors in photoreceptors and glia

**Fig. 2:**
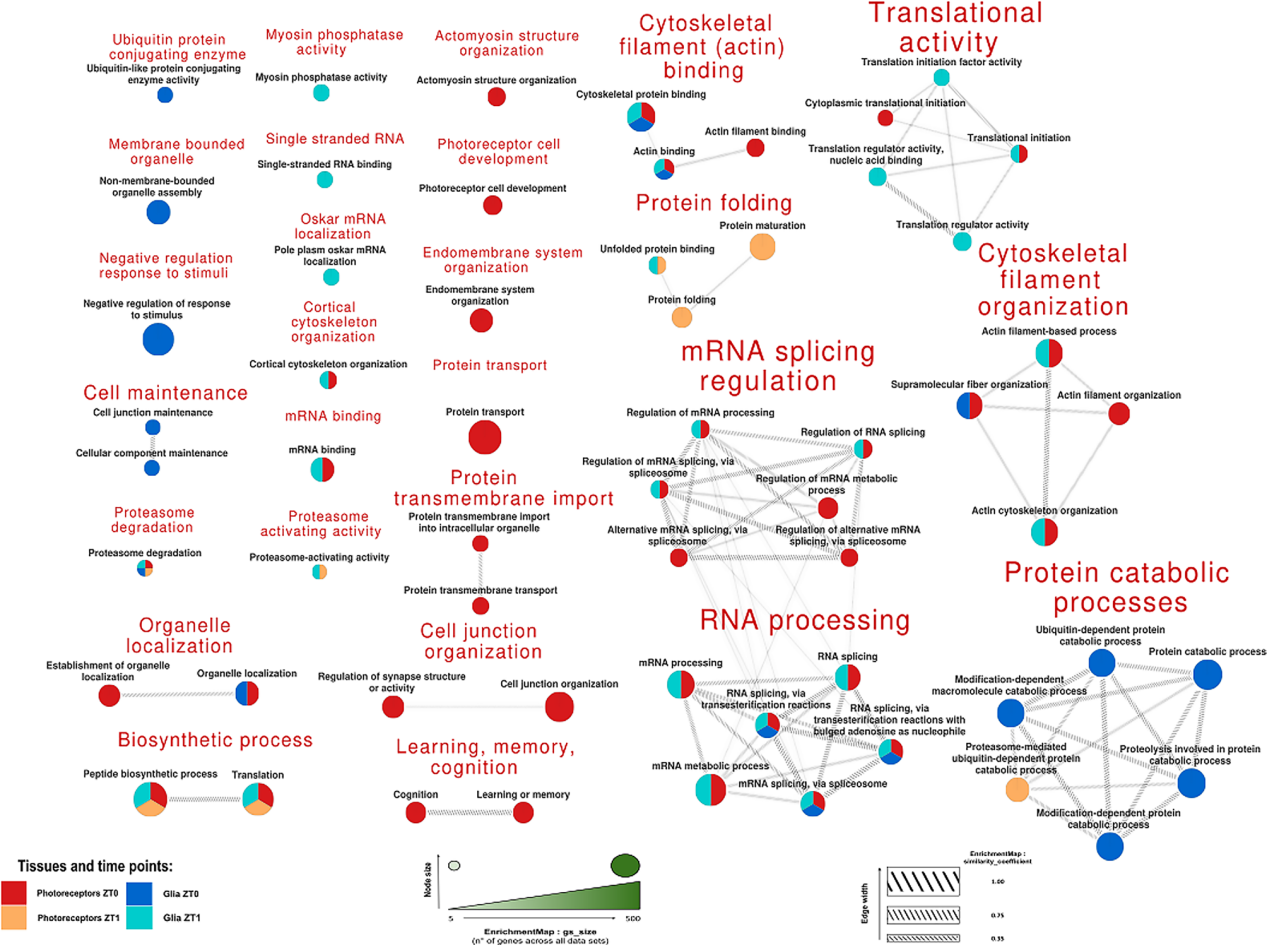
Enriched terms with relaxed (FDR q-values of 0.05) filtering criteria. Comparative visualization of the enriched terms, with the terms displayed as cake plots. The colored segments within each circle represent the datasets in which each term is enriched, and the cake size reflects the number of proteins associated with each term. The lines connecting the circles indicate the similarity between terms, with thicker lines representing stronger connections. Each group of terms is summarized by a general title in red based on functional or thematic categories. The proteins associated with each enriched term are provided in Supplementary Table S4. The filter was applied at an FDR q-value = 0.05

**Fig. 3:**
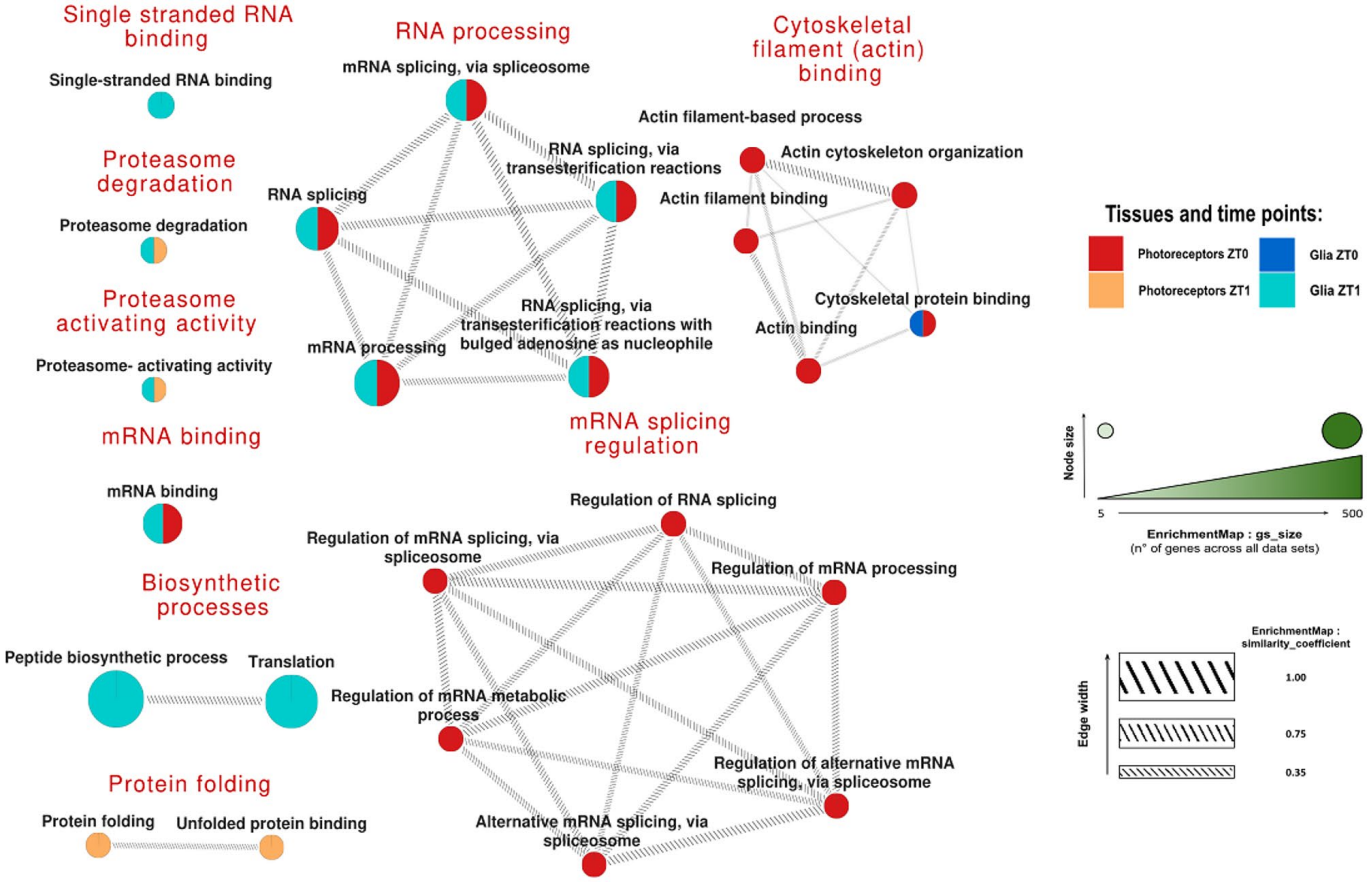
Enriched terms with stringent (FDR q-values of 0.001) filtering criteria. Comparative visualization of the enriched terms, with the terms displayed as cake plots. The colored segments within each circle represent the datasets in which each term is enriched, and the cake size reflects the number of proteins associated with each term. The lines connecting the circles indicate the similarity between terms, with thicker lines representing stronger connections. Each group of terms is summarized by a general title in red based on functional or thematic categories. The proteins associated with each enriched term are provided in Supplementary Table S5. The filter was applied at an FDR q-value = 0.001 to highlight the most significant enrichments

After constructing stable and reliable protein-protein interaction networks, we aimed to elucidate the functions of these proteins and identify which biological pathways were over- or under-represented among the dCRY interactors in the two neuronal populations under study, across the two experimental conditions. Enrichment analysis was performed using g: Profiler [47] focusing on Gene Ontology (GO). The findings are shown below, with both relaxed (Fig. 2) and stringent (Fig. 3) filtering criteria applied at FDR q-values of 0.05 and 0.001, respectively.

Figure 2 shows that certain terms are distinctly enriched in specific cellular populations or time periods. When we consider the terms enriched for a single dataset, we can associate specific cellular processes. Here, we observe that dCRY in photoreceptor cells in dark conditions binds proteins involved in protein transport, cell junction organization, and cytoskeleton management, but also in processes such as cognition, learning, and memory, as well as in the development of photoreceptor cells. On the other hand, when focusing on the unique results for proteins found in glial cells, we observe significant engagement in activities related to cell maintenance and protein catabolic processes at ZT0. In contrast, after 1 h of light exposure (ZT1), there seems to be a greater involvement in interactions with RNA molecules and cytoskeletal elements. However, several terms are still shared between populations. In this context, the application of a more stringent filter (FDR q-values of 0.001) proved highly beneficial in prioritizing the most reliable terms, represented in Fig. 3, some of which were already characteristic in Fig. 2.

Among them, we highlighted the involvement in protein folding activities for photoreceptors at the beginning of the day, while terms associated with protein degradation were shared with proteins detected in the glia after the lights-on, specifically in reference to the proteasome machinery; additionally, in this group, highly specific terms related to protein synthesis and translation emerged.

Moreover, with this increased stringency, the specific involvement of the Photoreceptors ZT0 population in mRNA splicing regulation and cytoskeleton organization was highlighted. Notably, the contrast between the Photoreceptors ZT0 and the Glia ZT1 groups regarding terms related to RNA splicing via transesterification suggests that these processes occur under opposite conditions in the two cellular populations. The recurrence of RNA-related terms across both filtering processes suggests that dCRY plays a different role in these processes within photoreceptors and glia.

### *Drosophila* CRY interactors associated with RNA-related terms

**Fig. 4:**
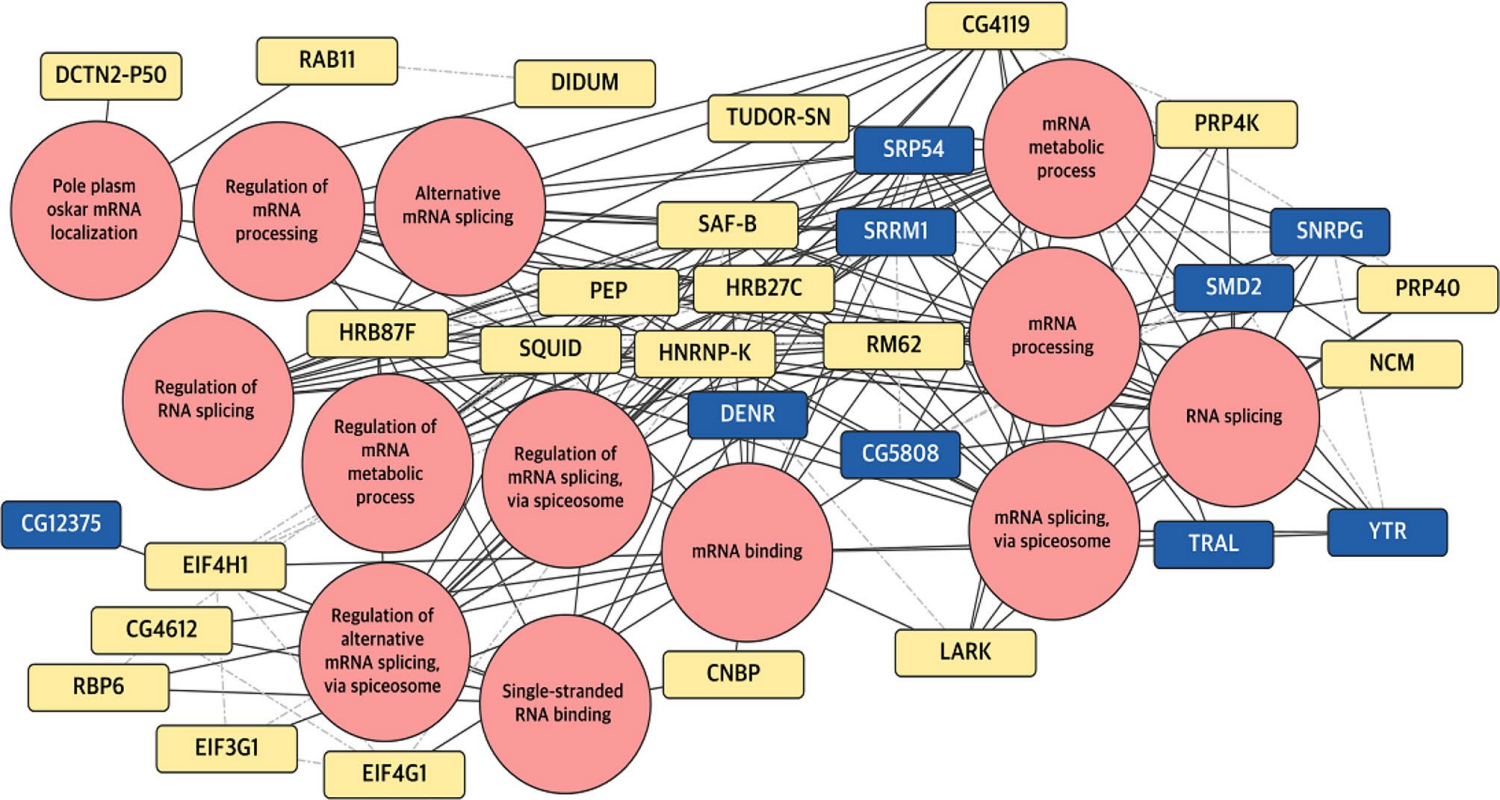
Protein Interaction Networks (PINs) of proteins derived from glial cells annotated with RNA terms relationships. This network presents the population of proteins forming complexes with dCRY in glial cells. In rose are shown the enriched terms related to RNA processing connected with black lines to the specific proteins. Grey dash-dot lines connect proteins to each other, representing protein-protein interactions. The protein nodes are colored based on the time point when the sample was collected, yellow for ZT1 (light-activated complexes) and blue for ZT0 (light-independent complexes)

The “terms-based networks” (Figs. 2 and 3) served as the starting point for integrating the previously constructed PPINs using STRINGapp, with the annotation generated by the enrichment process. This approach enabled us to evaluate the specific content of these terms and determine their dependency on light. In this section we highlight proteins enriched in RNA-related terms which encompass various mechanisms involved in RNA manipulation and processing, crucial for subsequent steps in protein translation. The resulting networks are illustrated in Figs. 4 (glial cells) and 5 (photoreceptor cells), and tables listing the proteins associated with each term are provided in the supplementary materials (Supplementary Tables S6 and S7).

Figure 4 presents the Protein Interaction Network (PIN) updated with the term annotations for proteins detected in glia. This visualization is derived from a more comprehensive dataset consisting of 219 proteins, of which 48 and 75 are specifically detected in darkness and light, respectively, across 56 terms (Supplementary Table S6). In the represented network, 13 RNA-related terms are linked to 9 darkness-specific and 21 light-specific complexes with dCRY

**Fig. 5:**
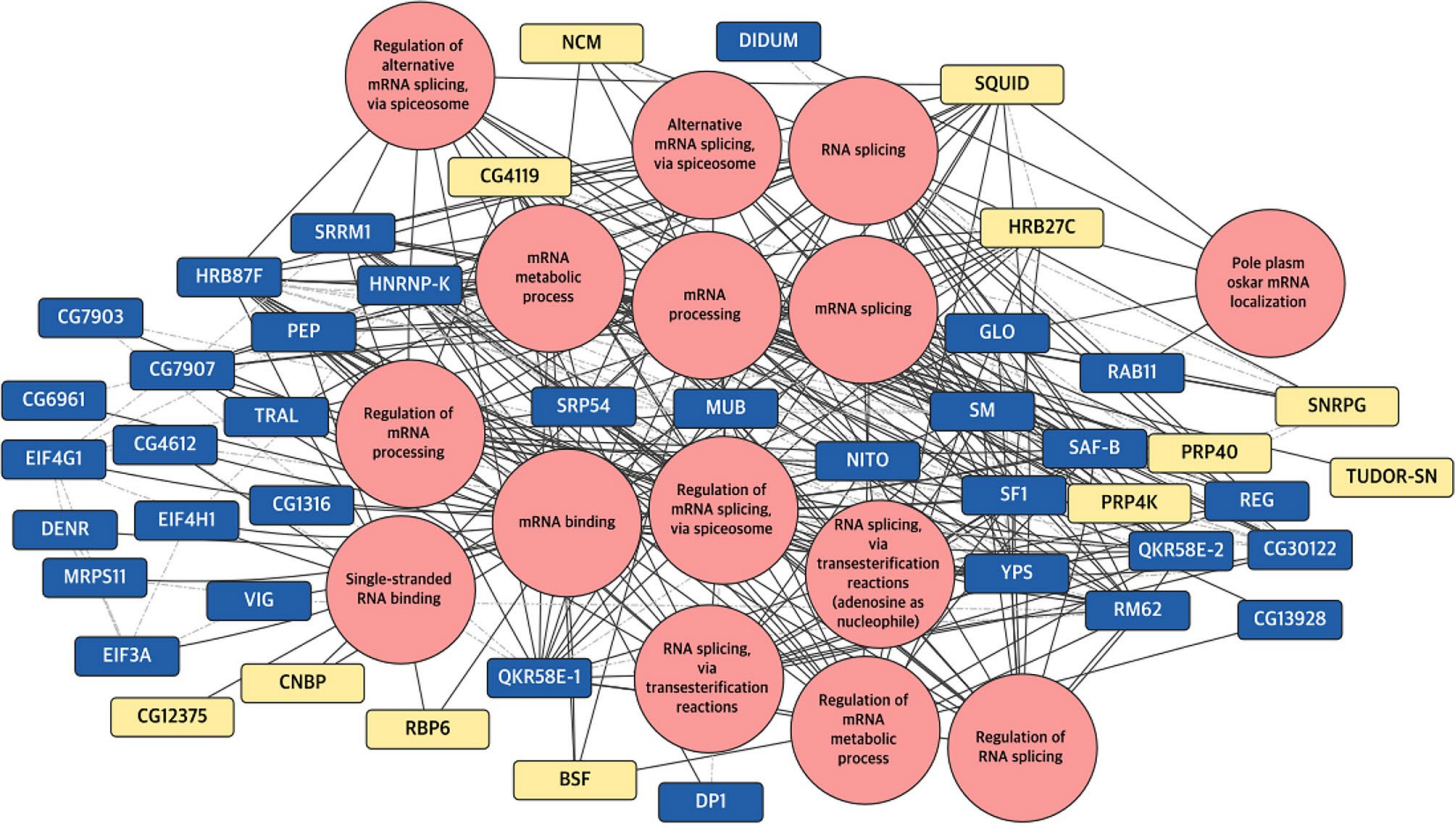
Protein Interaction Networks (PINs) of proteins derived from photoreceptor cells annotated with RNA terms relationships. This network presents proteins which form complexes with dCRY in photoreceptor cells. In rose are shown the enriched terms related to RNA processing connected with black lines to the specific proteins. Grey dash-dot lines connect proteins to each other, representing protein-protein interactions. The protein nodes are colored based on the time point when the sample was collected, yellow for ZT1 (light-activated complexes) and blue for ZT0 (light-independent complexes)

The full-term network for proteins detected in photoreceptors consists of 59 terms and 288 proteins, with 142 and 61 proteins specifically detected in darkness and light conditions, respectively (Supplementary Table S7). In Fig. 5, 15 terms and 48 proteins are displayed, with 33 specifically detected in darkness and 12 after light exposure.

These graphs illustrate contrasting patterns of dCRY complexes formation between the two cell populations. Specifically, in glial cells, there is a pronounced formation of complexes with proteins involved in RNA processing upon light exposure (21 proteins), whereas in retinal cells the majority of interactions occur in the darkness (33 proteins). These findings suggest a tissue- and time-specific dCRY-dependent remodelling of the proteome involved in RNA biology, highlighting distinct protein dynamics between the two tissues at different time points.

### A case study: SQUID

**Fig. 6:**
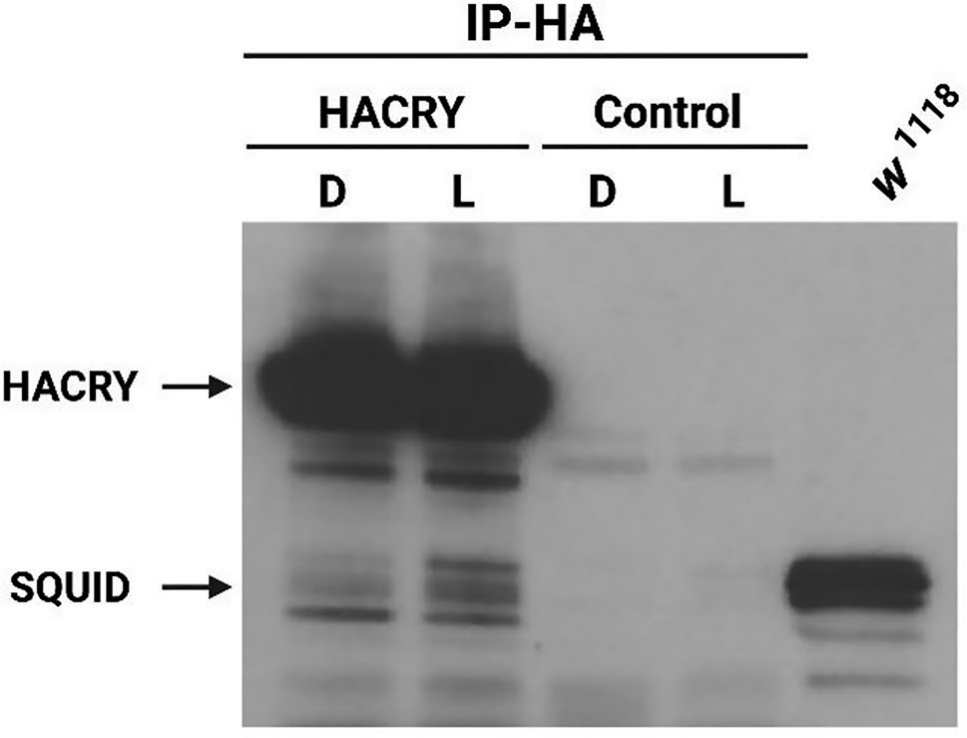
dCRY interacts with SQUID in TIM-expressing cells. HACRY-overexpressing flies (*tim*-GAL4/+;*UAS-HAcry*/+) and relative control (*tim*-GAL4) were reared in 12:12 LD and collected in the dark (ZT0, “D”) and in the light (ZT0 + 15 min light pulse, “L”). Membranes were probed with anti-SQUID and anti-HA antibodies. *w*^*1118*^ flies reared in 12:12 LD and collected at ZT1 were used as positive control of the antibody

The RNA-binding protein SQUID is a key component of ribonucleosomes, complexes composed of RNA and proteins [43, 52]. SQUID is one of the most reported proteins, being involved in 14 out of 15 enriched terms in the retina (Fig. 5), along with PEP, HRB87F, and HRB27C. However, only SQUID and HRB27C formed complexes with dCRY in both tissues after light exposure (Figs. 4 and 5). We have previously performed Co-IP and MS analysis from heads of transgenic flies expressing HACRY in TIM-positive cells [18, 53]. In these experiments, a 40-kDa species was specifically observed in the sample (either at ZT0 and after a 15 min light-pulse given at the same ZT), that was identified as SQUID (Supplementary Table S8). A Western blot with an anti-SQUID antibody revealed several bands of the expected molecular weight (around 36 kDa) that can be ascribed to the different SQUID isoforms, specifically in the samples where HACRY is present, and not in the controls (flies from the *tim*-Gal4 driver collected at the same time-points, while *w*^*1118*^ flies were used as a control for the antibody), demonstrating an interaction between the two proteins either in the dark and after 15 min of light exposure (Fig. 6).

#### Squid affects circadian locomotor activity

**Fig. 7:**
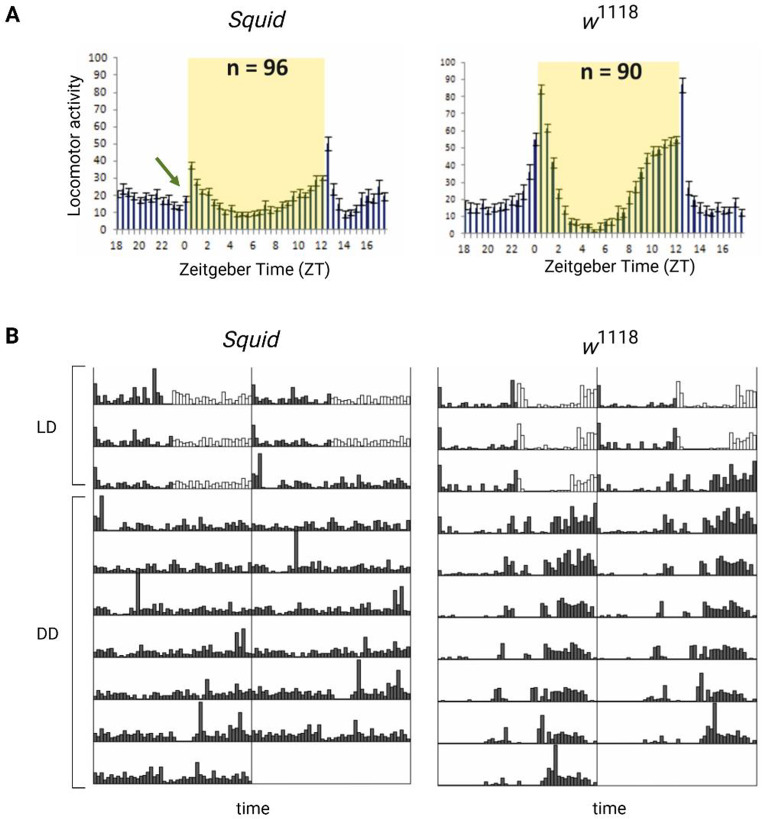
Locomotor activity of *Squid* and *w*^*1118*^ control flies. (**A**) Activity means ± SEM of flies over three days of LD cycles (n: number of flies). The yellow shading indicates the light phase. The green arrow indicates the lack of morning anticipation in *Squid* mutants. On the Y axis the number of recorded events in 30 min bins are reported. (**B**) Representative double-plotted locomotor activity of a single fly recorded for 3 days under LD and 7 days under DD conditions (black bars: dark phase; white bars: light phase)

We decided to investigate a possible role for *Squid* in the circadian machinery using a mutant line generated by P-element insertion in the first intron of the gene, that highly reduces its expression [41–44]. Flies were entrained for 3 days in light-dark (LD) cycles at constant temperature (23 °C) followed by 7 days of constant darkness (DD). The *Squid* mutant exhibited an impairment of the locomotor behavior (Fig. 7; Table 2): in LD conditions flies presented the canonical bimodal profile, but a severe loss of the morning anticipation of the locomotor activity was displayed (Fig. 7A; Table 2). A high percentage of flies also showed an arrhythmic behavior in DD, although no defects were monitored concerning the period, which was comparable to *w*^*1118*^ controls (Fig. 7B; Table 2).

**Table 2:** Locomotor activity of *Squid* and *w*^*1118*^ control flies

Genotype	Number of flies (*n*)	Alive (*n*)	Rhythmic (*n*)	Rhythmicity (%)	Period τ (h)	SEM	Morning Onset (%)	Evening Onset (%)	Morning Index	SEM
*w* ^*1118*^	90	77	64	83,1	23,8	0,05	72,7	98,7	0,14	0,02
*Squid*	96	80	3	3,8***	23,9	0,04	21,3***	72,5	0,02***	0,02

Summary of locomotor activity data, derived from three independent biological replicates. *Squid* mutants vs. *w*^*1118*^ control flies, two-way ANOVA, Bonferroni post hoc test. ***P < 0.001; **P < 0.01; *P < 0.05

An analysis of PDF-positive cells (l-LNv and s-LNv clock neurons) [54, 55] revealed that PDF projections appear highly disorganized in *Squid* mutant compared to control flies (Supplementary Figure S3), suggesting that the altered pattern of this released neuropeptide could, at least partially, account for the defects in the locomotor activity observed in the *Squid* mutant.

## Discussion

*Drosophila* CRYPTOCHROME (dCRY) is involved in many different processes, from photoreception and circadian clock entrainment [1–4] to regulation of synaptic plasticity [20, 21] and magnetoreception [26–30]. Its circadian function is strictly connected with light: indeed, after light exposure it undergoes a conformational change, is activated [11, 12] and may bind different proteins. However, dCRY accumulates during the night, reaching high levels before the lights-on: in the dark it forms complexes with many proteins [18, 22, 31, 53], indicating that its role is just as significant during the dark phase as it is during the day. Considering the structure of dCRY, which features multiple PPI domains and a series of evolutionarily conserved linear motifs, mostly located in its CCT [10, 18], it is reasonable to assume that a number of dCRY interactors, along with potential uncharacterized functions, have yet to be identified and further investigated.

Recently, a comprehensive analysis of its interactome in S2 cells has been published, uncovering several novel interactors of dCRY in vitro [39]. In this work, using an experimental approach based on co-immunoprecipitation (Co-IP) followed by mass spectrometry (MS) analysis, we extended such investigation obtaining the in vivo interactome of dCRY in two different cell populations – retina photoreceptors and glial cells. To follow the functional dynamics of interactions, samples were collected at two time points, just before lights-on (ZT0) and 1 h after lights-on (ZT1). In order to identify proteins forming complexes with dCRY in a tissue- and/or time-specific manner, we summarized our network composition representing the number of shared interactors between the Photoreceptors dataset at ZT0 and ZT1, the Glia dataset at ZT0 and ZT1, and both datasets at both time points.

### Diverse tissue- and time-specific roles of dCRY interactors

The finding that the dCRY interactome in the photoreceptors is richer at ZT0 than ZT1 hints for important roles in the darkness and is in accordance with our previous observations [18, 19, 22]. A distinct scenario was noted in glial cells, where an increased number of proteins within the dCRY interactome were identified following light exposure.

In Fig. 2 (relaxed filtering criteria, FDR q-values of 0.05), despite several terms being still shared between populations, a number of tissue- and time-specific terms emerged. Importantly, most of these results align with existing scientific literature.

By way of example, looking at the term “Learning, memory, cognition” enriched in the Photoreceptors dataset, it is well known that light conditions are able to positively and negatively modulate memory processes [56–59], with light being essential in long-term memory maintenance [59]. In this context dCRY has recently been shown to have a prominent role, since knock-out flies exhibit five-day memory impairment [60].

In addition, it is also known that retinal gap junctions are modulated by light [61–64]: indeed, in the mammalian retina the opening of gap junctions, which increases their conductance, was reported to be more frequent during darkness [65]. This is in accordance with the enrichment of the “Cell junction organization” term in the Photoreceptors dataset at ZT0.

In Fig. 3 (stringent filtering criteria, FDR q-values of 0.001), only the most reliable terms were retained, providing further insights into the tissue- and time-specificity of the biological processes involving dCRY interactors.

We observed that proteasome-related proteins are mostly present after light exposure, but not during the darkness. This is entirely consistent with dCRY’s role in mediating the light-dependent degradation of proteins like TIMELESS [13] and BRP [19]. Furthermore, in the mammalian retina light acts directly on photoreceptors to decrease melatonin synthesis and protein levels by a mechanism involving proteasomal degradation, and in general a sudden increase in light intensity leads to a marked rise in the number of autophagic vacuoles [66]. In addition, the removal of excess rhodopsin through ubiquitination can serve as a protective mechanism against light-induced retinal damage [66], and retinal degeneration in mice can be delayed by increasing photoreceptor proteasomal activity [67].

The “peptide biosynthetic process” term enrichment in the Glia dataset at ZT1 might be related to the glial contribution to the protein synthesis occurring in the α/β neurons of the mushroom bodies after light exposure in accordance with the model proposed by Inami and Sakai [60]. Indeed, while providing metabolic support to neurons has always been recognized as one of the major functions of glial cells, in recent times a deterministic role of glia metabolism and signaling on brain function and behavior is emerging, in particular in the mushroom bodies [68–70].

The increased stringency has highlighted the specific involvement of the Photoreceptors dataset at ZT0 in mRNA splicing regulation, detailed below, and cytoskeleton organization. In the *Drosophila* retina, dCRY is present in the rhabdomeres of all photoreceptor cells and is able to interact with F-actin [22]; moreover, in other organisms, significant changes associated with actin cytoskeleton occur in retinal photoreceptors and pigment epithelium in the dark [71–74].

### dCRY may contribute to the spatiotemporal regulation of RNA metabolism

Light and darkness have been extensively shown to regulate alternative splicing in plants [75, 76]. This process is also regulated by the circadian clock, both in plants [77] and animals [78, 79]. In higher organisms, alternative splicing plays a crucial role in neuronal tissues, in particular in the retina, which displays some of the highest levels of differentially spliced genes in the body [80]. This, together with the fact that several genes in mouse retina show the highest or the lowest expression levels near the end of the light phase [81], makes our data of potential interest to further investigate the roles exerted by dCRY and its interactors in the process of RNA and in particular mRNA metabolism at different time points.

To this end, we integrated the previously constructed PPINs with the annotation generated by the enrichment process, highlighting the proteins enriched in RNA-related terms in the two tissues and the time point when the sample was collected.

Among them, we decided to further characterize the role of the RNA-binding protein SQUID, which was found to be involved 14 out of 15 enriched terms in the retina and to interact with dCRY in both retina and glia after light exposure.

### The hnRNP SQUID bridges RNA metabolism and circadian clock

SQUID belongs to the hnRNP A family of RNA binding proteins and is a key component of ribonucleosomes, essential for various cellular processes involved in the regulation of RNA metabolism, including its transcription, processing, transport, and degradation [43]. It was previously shown that SQUID can bind retinoblastoma tumor suppressor RBF [82] or HRB27C [83] and regulate splicing. Its function is crucial for normal cell development, and its disruption may relate to the development of neurodegenerative disorders, such as Fragile X-associated tremor/ataxia syndrome [84], Huntington’s disease [85] or Alzheimer’s disease [86].

In the context of SQUID interacting with dCRY, ribonucleosome involvement suggests that SQUID likely contributes to the rhythmic regulation of RNA through its structural and regulatory roles in these complexes. This regulation occurs at multiple levels, including post-transcriptional, transcriptional, translational, and post-translational processes, thereby affecting the overall dynamics of RNA within the cell.

We focused on the possible role of *Squid* in the circadian machinery, and our detailed analysis showed that it is connected with pacemaker function. We observed that *Squid* mutant flies exhibited an impairment of their locomotor behavior, with a severe loss of the morning anticipation in LD and, in high percentages, an arrhythmic behavior in DD. This behavioral change is reminiscent of the clock mutant *per*^*0*^ [87], suggesting that the pacemaker function is impaired in these flies. Trying to understand this phenomenon we analyzed the morphology of the main clock neurons, PDF-expressing cells, and we found abnormal arborization of LNvs terminals, with high levels of disorganization. This could be the result of developmental defects during dendrite morphogenesis, which was described in *Squid* mutants in sensory neurons [88].

## Conclusions

In conclusion, our work provides the first tissue- and time-specific dCRY interactome. Our data are consistent with the current scientific literature, but at the same time provide valuable insights into still unknown biological processes in which this multifaceted photoreceptor can be involved.

We have revealed a potential role of dCRY in the circadian regulation of RNA metabolism. Considering the growing body of evidence that supports RNA disturbances as a contributing factor to various neurodegenerative disorders, along with the dysfunction of hnRNPs linked to numerous neurological defects, we believe that our research provides significant insights into the relationship between the circadian clock, RNA metabolism, and neurodegenerative diseases.

## Supplementary Information

Below is the link to the electronic supplementary material.

Supplementary Material 1

Supplementary Material 2

Supplementary Material 3

Supplementary Material 4

Supplementary Material 5

Supplementary Material 6

Supplementary Material 7

Supplementary Material 8

Supplementary Material 9

Supplementary Material 10

Supplementary Material 11

Supplementary Material 12

Supplementary Material 13

Supplementary Material 14

Supplementary Material 15

Supplementary Material 16

Supplementary Material 17

Supplementary Material 18

Supplementary Material 19

## Data Availability

MS data are submitted as supplemental data.

## Abbreviations

BRP: Bruchpilot
CLK: Clock
Co-IP: Co-immunoprecipitation
CRY: Cryptochrome
CTT: C-terminus tail
CYC: Cycle
DD: Constant darkness
FDR: False discovery rate
GO: Gene ontology
HA: Hemagglutinin
LC: Liquid chromatography
LD: Light-dark
l-LNv: Large ventrolateral neurons
MF: Molecular functions
MMTS: Methyl methanethiosulfonate
MS: Mass spectrometry
PER: Period
PHR: Photolyase homology region
PIN: Protein interaction network
PPI: Protein-protein interaction
PPIN: Protein-protein interaction network
s-LNv: Small ventrolateral neurons
TCEP: Tris(2-carboxyethyl)phosphine
TIM: Timeless
ZT: *Zeitgeber* time
