# Supplementary material for: Cryptochrome interaction networks across different tissues in Drosophila melanogaster
Source: Biol Direct. 2025 Nov 28;20:114. doi: 10.1186/s13062-025-00696-x (PMC12661795; doi:10.1186/s13062-025-00696-x)
Supplement: Supplementary file 1 — Supplementary Material 1 [file 13062_2025_696_MOESM1_ESM.docx]

**SUPPLEMENTARY**

**File Content:**

- **Supplementary Methods**: additional information about Co-IP and MS protocols.
- **Supplementary Table S1**: table, title, and caption.
- **Supplementary Figure S1**: title and caption.
- **Supplementary Figure S2**: title and caption.
- **Supplementary Table S2**: title and caption.
- **Supplementary Table S3**: title and caption.
- **Supplementary Table S4**: title and caption.
- **Supplementary Table S5**: title and caption.
- **Supplementary Table S6**: title and caption.
- **Supplementary Table S7**: title and caption.
- **Supplementary Table S8**: title and caption.
- **Supplementary Figure S3**: figure, title, and caption.

**Supplementary Methods**

Co-Immunoprecipitation

Proteins from 50 males’ heads were extracted mechanically (using motor-operated micro-pestles) in 50 µl of extraction buffer (20 mM HEPES, 100 mM KCl, 2.5 mM EDTA, 5% glycerol, 0.5% Triton X-100, 1 mM DTT, complete protease inhibitors) (Roche). The extracts were cleared by centrifugation (1 hour at full speed in a microcentrifuge at 4ºC) and the supernatants were moved into new 1.5 ml tubes. Immunocomplexes were precipitated by Pierce™ Anti-HA Magnetic Beads (Thermo Fisher Scientific) following the manufacturer’s instructions. Immunoprecipitation reactions were carried out at 4°C overnight. After washing and elution, the immuno-complexes were analyzed by Mass Spectrometry.

Protein identification by Mass Spectrometry

Beads with bounded proteins were suspended in dissolution buffer (100 mM NH_4_HCO_3_). First, the cysteines were reduced by 1 hour incubation with 5 mM tris(2-carboxyethyl)phosphine (TCEP) at 60^°^C followed by 10 min incubation at room temperature with 20 mM methyl methanethiosulfonate (MMTS). Digestion was provided at 37°C overnight with 1 µg of trypsin (Promega). After digestion, peptides were dried in SpeedVac and re-suspended in 10 µl of extraction buffer (0.1% TFA, 2% acetonitrile) with sonication. The next step was processed using single-pot solid-phase-enhanced sample preparation (SP3). Magnetic beads mix were prepared by combining equal parts of Sera-Mag Carboxyl hydrophilic and hydrophobic particles (09-981-121 and 09-981-123, GE HealthCare). The beads mix was washed three times with MS-grade water and re-suspended in a working concentration of 10 µg/µl. The beads mix was then added to the samples and then suspended in 100% acetonitrile; this step was repeated 3 times. Pure peptides were eluted from the beads by using 2% acetonitrile in MS-grade water. Using a magnet, the peptides solution was separated from beads. Peptide mixture was dried in SpeedVac and re-suspended in 80 µl extraction buffer (0.1% TFA, 2% acetonitrile) with sonication.

Samples were analyzed using LC-MS system composed of Evosep One (Evosep Biosystems) coupled to an Orbitrap Exploris 480 mass spectrometer (Thermo Fisher Scientific) via Flex nanoESI ion source (Thermo Fisher Scientific). Samples were loaded onto disposable Evotips C18 trap columns (Evosep Biosystems) according to the manufacturer protocol with minor modifications. Briefly, Evotips were activated with 25 µl of 80% solution solvent B and 20% solvent A by 1 min centrifugation at 600 g followed by 2 min incubation in 2-propanol. After equilibration with 25 µl of solvent A, 20 µl of each sample solution was loaded onto the solid phase. Bound peptides were washed with 50 µl and covered with 200 µl of solvent A. Chromatography was carried out at a flow rate 250 nl/min using the 88 min (15 samples per day) preformed gradient on EV1106 analytical column (Dr Maisch C18 AQ, 1.9 µm beads, 150 µm ID, 15 cm long; Evosep Biosystems). Data was acquired in positive mode with a data-dependent method using the following parameters. MS1 resolution was set at 60,000 with a normalized AGC target 300%, Auto maximum inject time and a scan range of 300 to 1600 m/z. For MS2, resolution was set at 15 000 with a Standard normalized AGC target, Auto maximum inject time and top 40 precursors within an isolation window of 1.6 m/z considered for MS/MS analysis. Dynamic exclusion was set at 20 s with allowed mass tolerance of ± 10 ppm and the precursor intensity threshold at 5e3. Precursors were fragmented in HCD mode with normalized collision energy of 30%. Spray voltage was set to 2.1 kV, funnel RF level at 40, and heated capillary temperature at 275 °C.

MS/MS data were pre-processed with the Mascot Distiller software (v. 2.4.2.0; Matrix Science), then obtained peptide masses and fragmentation spectra were matched to the UniProt database (Sprot: 569,516 sequences; 205,866,895 residues; Trembl: 249,308,459 sequences; 86,853,323,495 residues) with taxonomy filter *Drosophila melanogaster* (42,762 sequences) using the Mascot search engine (Mascot Daemon v. 2.4.0, Mascot Server v. 2.4.1, and Matrix Science). To reduce mass errors, the peptide and fragment mass tolerance settings were established separately for individual LC-MS/MS runs after a measured mass recalibration, resulting in values 5 ppm for parent and 0.01 Da for fragment ions. The rest of search parameters were as follows: enzyme specificity was set to trypsin, methylation of cysteine was set as fixed and an oxidation of methionine was set as a variable modification. The protein mass was left as unrestricted and mass values as monoisotopic with two missed cleavages being allowed. The mass calibration and data filtering were carried out with MScan software (http://proteom.ibb.waw.pl/mscan/). The Decoy Mascot functionality was used for keeping FDR for peptide identifications below 1%. All peptides with q-values > 0.01 and proteins identified by a subset of peptides from another protein were removed from further analysis. The list of identified proteins was exported to Excel MS software.

|  | **ZT0** | **ZT1** |
| --- | --- | --- |
| **Mentioned in the paper**  **+** | both = 0.5  APEX = 0.25  TID = 0.25 | both = 0.5  APEX = 0.25  TID = 0.25 |
| **Significant**  **+** | both = 1  APEX = 0.5  TID = 0.5 | both = 1  APEX = 0.5  TID = 0.5 |
| **Highly significant**  $\times$ | both = 2  APEX = 1  TID = 1 | both = 2  APEX = 1  TID = 1 |
| **ZT0/1 = 1**  or  **both = 0.5** | **DARK**  **score** | **LIGHT**  **score** |

**Supplementary Table S1. Scoring matrix for protein list comparisons.** In order to compare the method used in this study and the new approach described in Ozcelik *et al*. (2024), protein lists were generated using two techniques, APEX and TurboID, with considerations for the effects of dark and light conditions. Indeed, Ozcelik and colleagues employed proximity-dependent biotinylation techniques, combining engineered BioID (TurboID) and APEX (APEX2) enzymes with mass spectrometry to identify *in vitro* dCRY interactors in *Drosophila* S2 cells. The development of the TurboID engineered biotin ligase in 2018 allowed for a reduction in incubation time, reaching saturation in just 10 minutes, making it highly suitable for studying short-lived interactions. Due to the high number of indirect interactors and/or neighboring proteins that do not physically interact with the fusion protein, they also incorporated results obtained through APEX2, an engineered soybean ascorbate peroxidase for proximity labeling, which reaches saturation in 30 minutes. The complementary use of these fast-acting enzymes reduced non-specific labeling and aimed to detect short-lived interactions more effectively. They categorized proteins as “significant” and “highly significant” based on the statistical reliability of protein binding: they applied the SAINT score (SAINT express algorithm determines the likelihood of each protein interacting with the bait protein) to evaluate the interactors with SAINT score ≥ 0.45 and ≥ 0.95 as “significant” and “highly significant”, respectively. By intersecting our lists with the data from their paper, we developed a simple scoring system, shown here, that explains the role of the proteins in the paper’s context and compares them to the experimental conditions applied in our study.

**Supplementary Figure S1. Photoreceptors-based networks.** The networks are colored according to the score. Specifically, the nodes in the network are colored in red if the proteins were identified with both techniques at ZT1 and in blue if the proteins were found at ZT0. Proteins that were not present in the Ozcelik *et al*. (2024) but were identified only with our technique, or proteins found under both light and dark conditions, are colored in white. In diamond shape, the proteins specifically found in the selected period; **A**) Photoreceptors ZT0: 36/185 proteins are mentioned in the paper, and 20/36 are “significant”. **B**) Photoreceptors ZT1: 25/83 proteins are mentioned in the paper, and 11/25 are “significant”.

**Supplementary Figure S2. Glia-based networks.** The networks are colored according to the score. Specifically, the nodes in the network are colored in red if the proteins were identified with both techniques at ZT1 and in blue if the proteins were found at ZT0. Proteins that were not present in the Ozcelik *et al*. (2024) but were identified only with our technique, or proteins found under both light and dark conditions, are colored in white. In diamond shape, the proteins specifically found in the selected period; **A**) Glia ZT0: 24/101 proteins are mentioned in the paper, and 12/24 are “significant”. **B**) Glia ZT1: 39/162 proteins are mentioned in the paper, and 16/39 are “significant”.

**Supplementary Table S2. Photoreceptors dataset and comparison with Ozcelik *et al*. (2024).**
Complete scoring matrix of the comparison between the Photoreceptors dataset and Ozcelik *et al*. (2024) data at ZT0 and ZT1. The “Score” column reports the values obtained by applying the scoring matrix described in Supplementary Table S1. Negative scores appear only for interactors identified in darkness (assigned for network visualization in Cytoscape, Supplementary Figure S1), whereas positive scores correspond to interactors identified in light. Proteins annotated as “both” were detected by both experimental approaches used by Ozcelik *et al*. (2024). “Significant proteins” are those reported in Ozcelik *et al*. (2024) as statistically supported interactors, defined using the SAINT express algorithm (SAINT score ≥ 0.45). The results are summarized in Table 1 and represented in Supplementary Figure S1.

**Supplementary Table S3. Glia dataset and comparison with Ozcelik *et al*. (2024).** Complete scoring matrix of the comparison between the Glia dataset and Ozcelik *et al*. (2024) data at ZT0 and ZT1. The “Score” column reports the values obtained by applying the scoring matrix described in Supplementary Table S1. Negative scores appear only for interactors identified in darkness (assigned for network visualization in Cytoscape, Supplementary Figure S2), whereas positive scores correspond to interactors identified in light. Proteins annotated as “both” were detected by both experimental approaches used by Ozcelik *et al*. (2024). “Significant proteins” are those reported in Ozcelik *et al*. (2024) as statistically supported interactors, defined using the SAINT express algorithm (SAINT score ≥ 0.45). The results are summarized in Table 1 and represented in Supplementary Figure S2.

**Supplementary Table S4. Proteins enriched in each term of Figure 2.** List of proteins enriched in each term obtained from the enrichment analysis performed using g:Profiler focusing on Gene Ontology (GO) represented in Figure 2. The filter was applied at an FDR q-value = 0.05.

**Supplementary Table S5. Proteins enriched in each term of Figure 3.** List of proteins enriched in each term obtained from the enrichment analysis performed using g:Profiler focusing on Gene Ontology (GO) represented in Figure 3. The filter was applied at an FDR q-value = 0.001.

**Supplementary Table S6. List of Glia proteins enriched in RNA-related terms retrieved from the previous analysis.** Here are listed the terms connected to Glia proteins, represented in Figure 4. The filter was applied at an FDR q-value = 0.05.

**Supplementary Table S7. List of Photoreceptors proteins enriched in RNA-related terms retrieved from the previous analysis.** Here are listed the terms connected to Photoreceptors proteins, represented in Figure 5. The filter was applied at an FDR q-value = 0.05.

**Supplementary Table S8. List of the proteins identified by LC-MS/MS.** For the electrophoretic bands, the identified proteins with their accession numbers and molecular masses are listed. Protein identification was performed with the MASCOT software searching LC-MS/MS data against the sequences of *Drosophila* of the SwissProt database. The fifth column contains a list of the peptides that were sequenced by MS/MS and that matched to the protein with a statistically significant score (P<0.05).


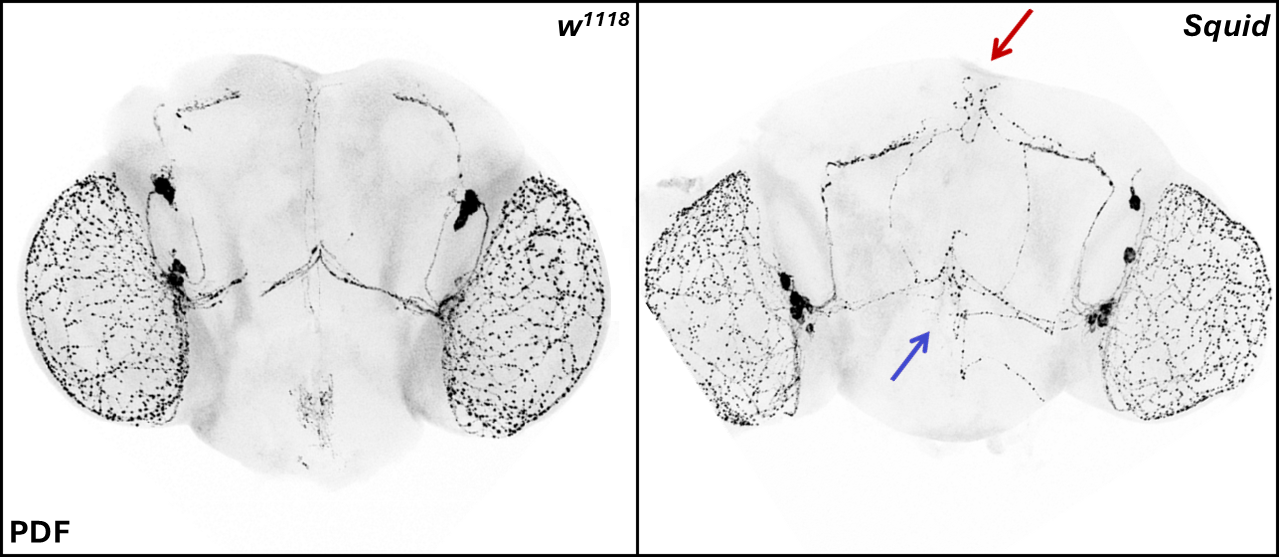


**Supplementary Figure S3. PDF-expressing cells network in *D. melanogaster* *w^1118^* control and *Squid* mutant adult brains.** In *Squid* mutant, abnormalities were observed in the posterior optic tract (POT), which innervates the ventral brain and forms bundles (indicated by the blue arrow) that are not observed in *w^1118^* control, with some projections linking the POT and small ventrolateral neurons (s-LNv) terminals in the dorsal brain. Moreover, s-LNv neurons, which normally send projections to the dorsal brain, exhibit additional arborizations and connections between the two hemispheres (indicated by the red arrow) in the *Squid* mutant that are absent in *w^1118^* control.
