# Supplementary figures and images for: Cryptochrome interaction networks across different tissues in Drosophila melanogaster
Source: Biol Direct. 2025 Nov 28;20:114. doi: 10.1186/s13062-025-00696-x (PMC12661795; doi:10.1186/s13062-025-00696-x)

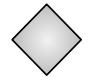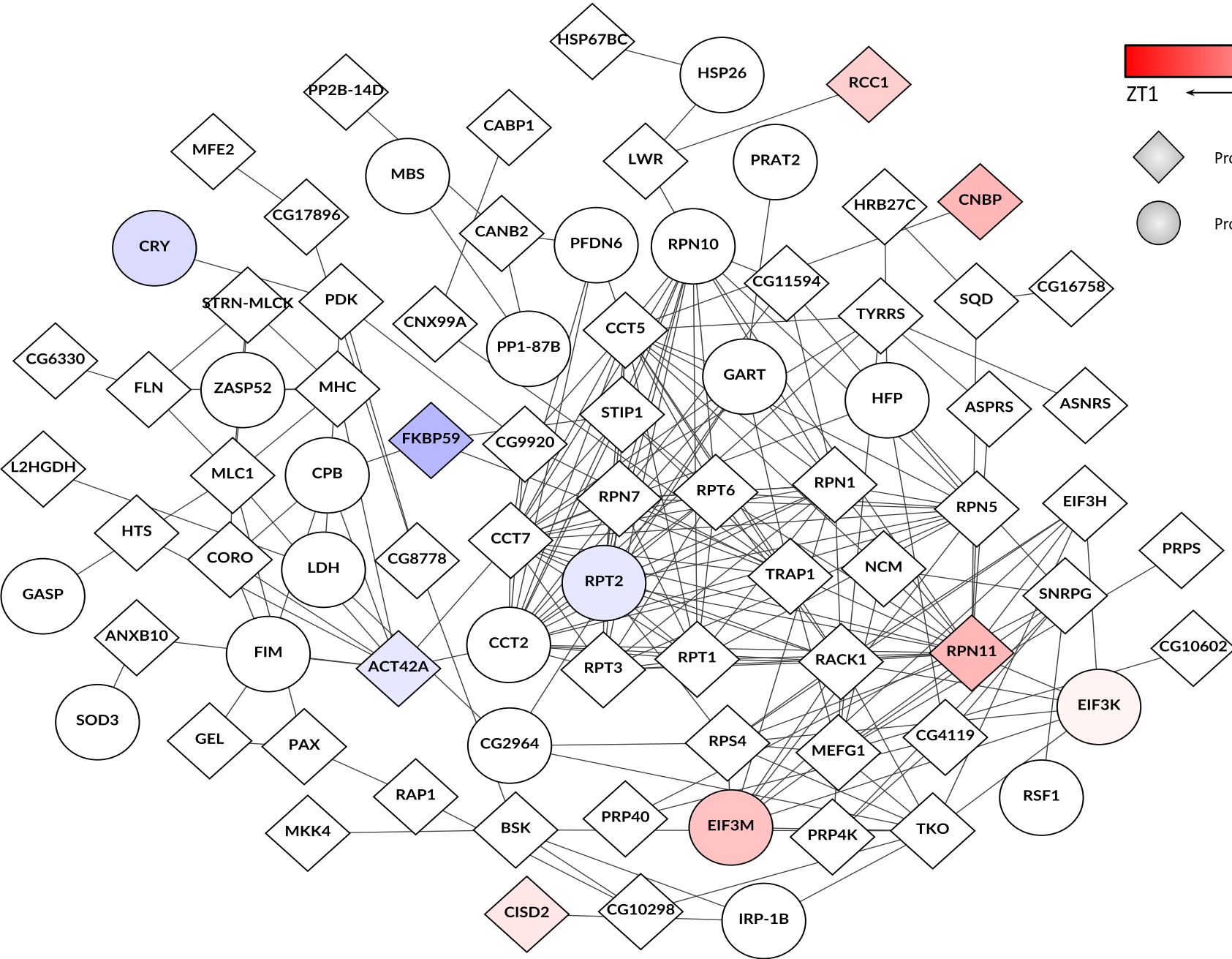

Supplement: Supplementary file 3 — Supplementary Material 3 [file 13062_2025_696_MOESM3_ESM.pdf]

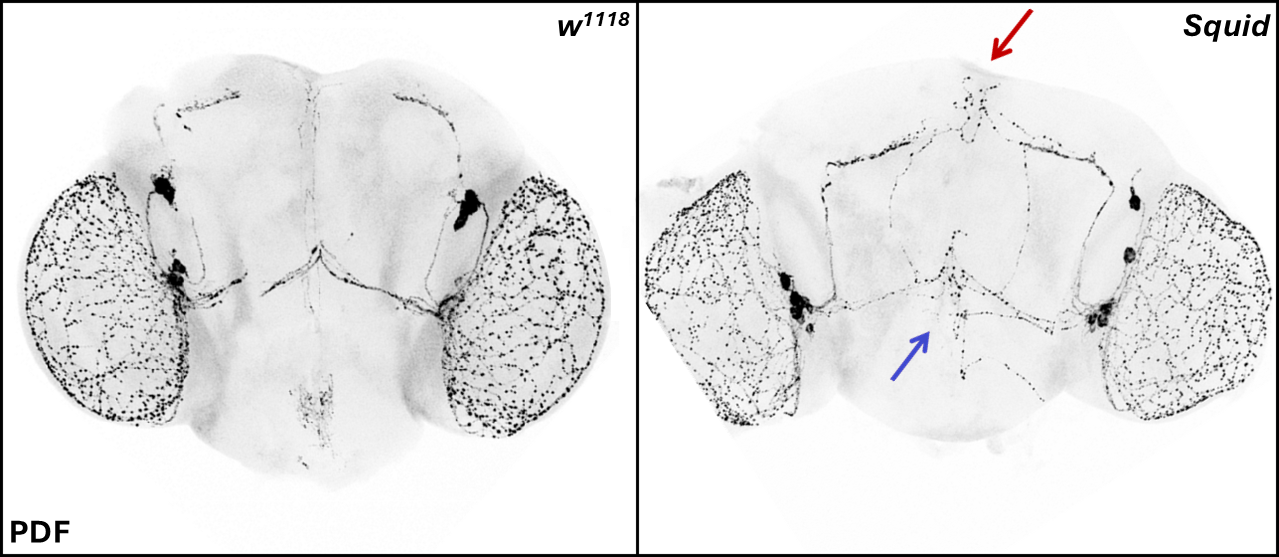

Supplement: Supplementary file 6 — Supplementary Material 6 [file 13062_2025_696_MOESM6_ESM.tif]
