# Supplementary material for: Cryptochrome interaction networks across different tissues in Drosophila melanogaster
Source: Biol Direct. 2025 Nov 28;20:114. doi: 10.1186/s13062-025-00696-x (PMC12661795; doi:10.1186/s13062-025-00696-x)
Supplement: Supplementary file 2 — Supplementary Material 2 [file 13062_2025_696_MOESM2_ESM.pdf]

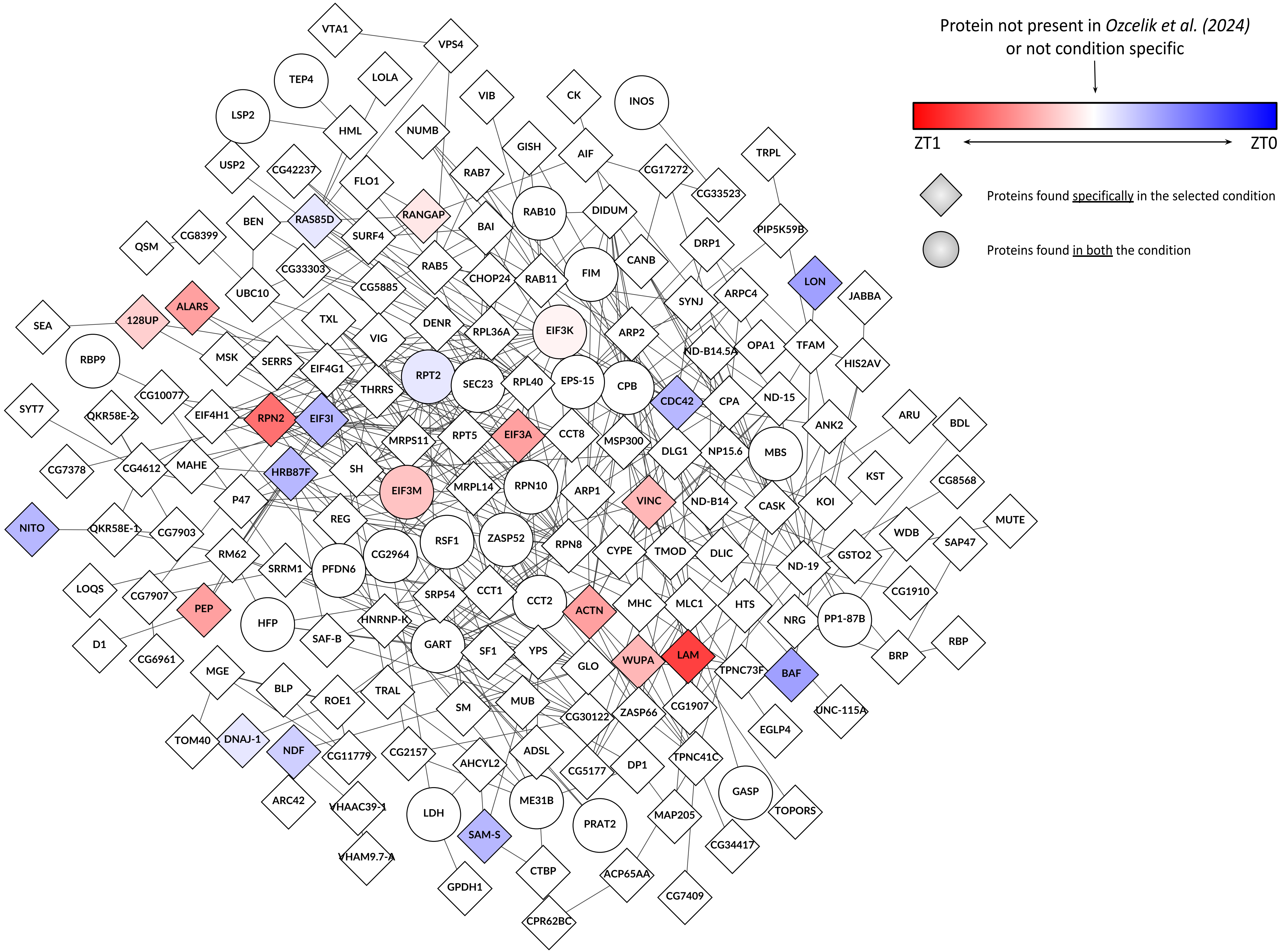

Protein not present in *Ozcelik et al. (2024)*
or not condition specific
ZT1
ZT0
Proteins found specifically in the selected condition
Proteins found in both the condition
VTA1
VPS4
TEP4
LOLA
LSP2
HML
VIB
CK
INOS
NUMB
GISH
AIF
USP2
CG42237
FLO1
RAB7
CG17272
TRPL
CG33523
RAS85D
RANGAP
RAB10
DIDUM
BEN
SURF4
BAI
PIP5K59B
QSM
CG8399
DRP1
CG33303
RAB5
FIM
CANB
LON
UBC10
CG5885
CHOP24
RAB11
SYNJ
ARPC4
JABBA
SEA
128UP
ALARS
TXL
DENR
RPL36A
EIF3K
ARP2
RBP9
MSK
SERRS
VIG
ND-B14.5A
OPA1
TFAM
HIS2AV
EIF4G1
RPT2
SEC23
RPL40
EPS-15
CPB
CG10077
THRRS
CDC42
CPA
ND-15
SYT7
QKR58E-2
EIF4H1
RPN2
EIF3I
ARU
BDL
MRPS11
RPT5
EIF3A
CCT8
MSP300
ANK2
DLG1
NP15.6
MBS
CG7378
CG4612
MAHE
HRB87F
SH
KST
CG8568
EIF3M
MRPL14
RPN10
ARP1
VINC
ND-B14
CASK
KOI
P47
REG
NITO
QKR58E-1
CG7903
MUTE
WDB
CG2964
RSF1
ZASP52
RPN8
SAP47
CYPE
TMOD
DLIC
GSTO2
RM62
SRRM1
PFDN6
ND-19
LOQS
CG1910
CG7907
SRP54
CCT1
CCT2
ACTN
MHC
MLC1
HTS
NRG
PP1-87B
PEP
HFP
HNRNP-K
SAF-B
D1
GART
SF1
YPS
GLO
WUPA
LAM
RBP
CG6961
BRP
TPNC73F
MGE
BAF
BLP
TRAL
ROE1
SM
MUB
CG30122
ZASP66
CG1907
UNC-115A
EGLP4
TOM40
DNAJ-1
NDF
CG11779
CG2157
AHCYL2
ADSL
CG5177
DP1
TPNC41C
ARC42
GASP
VHAAC39-1
ME31B
LDH
TOPORS
PRAT2
MAP205
SAM-S
CG34417
VHAM9.7-A
CTBP
GPDH1
ACP65AA
CG7409
CPR62BC
