# Supplementary material for: Cryptochrome interaction networks across different tissues in Drosophila melanogaster
Source: Biol Direct. 2025 Nov 28;20:114. doi: 10.1186/s13062-025-00696-x (PMC12661795; doi:10.1186/s13062-025-00696-x)
Supplement: Supplementary file 4 — Supplementary Material 4 [file 13062_2025_696_MOESM4_ESM.pdf]

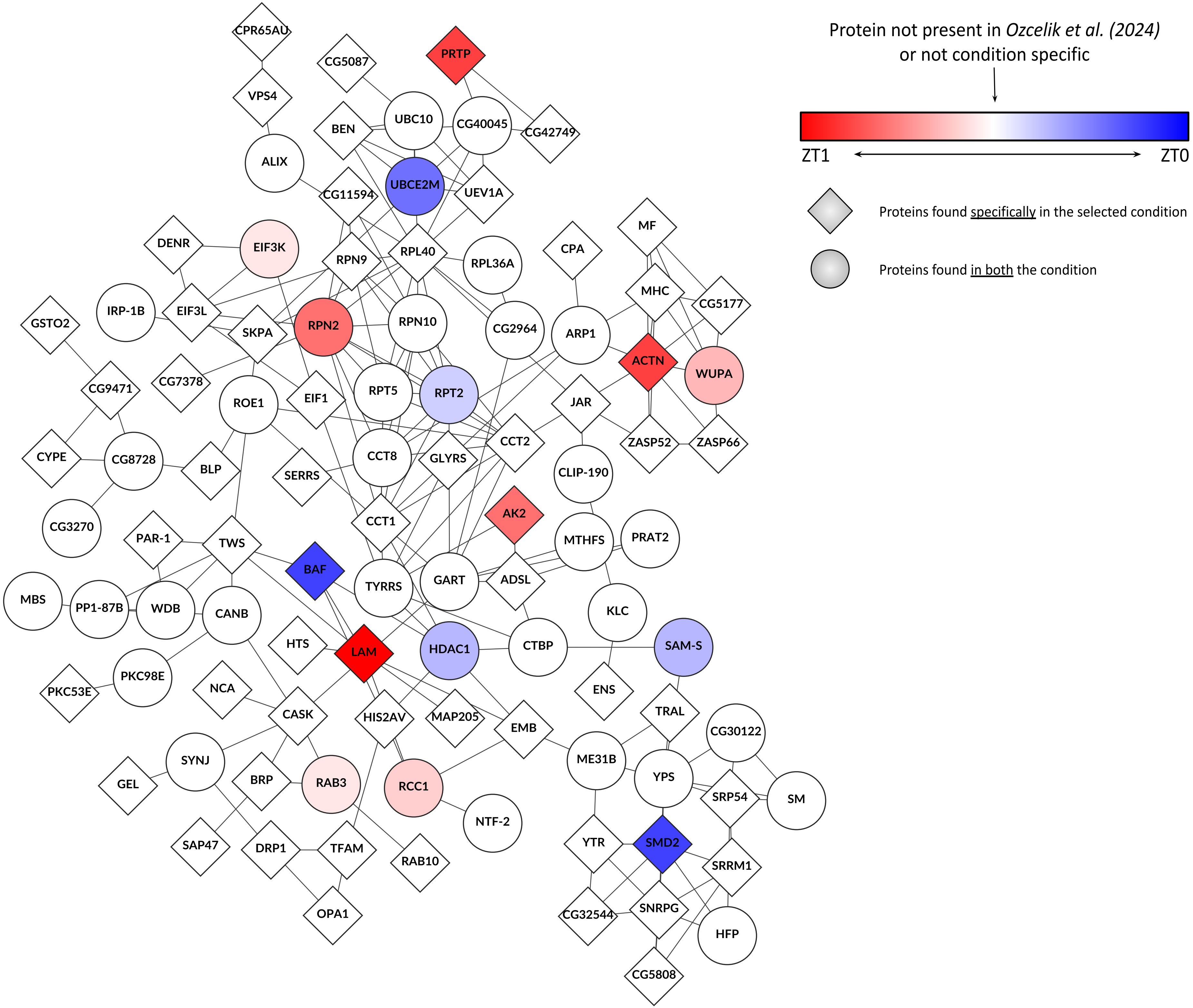
Protein not present in *Ozcelik et al. (2024)*
or not condition specific
ZT1
ZT0
Proteins found specifically in the selected condition
Proteins found in both the condition
CPR65AU
VPS4
ALIX
CG5087
PRTP
BEN
UBC10
CG40045
CG42749
CG11594
UBCE2M
UEV1A
DENR
EIF3K
RPN9
RPL40
RPL36A
CPA
MF
IRP-1B
EIF3L
GSTO2
SKPA
RPN2
RPN10
CG2964
ARP1
MHC
CG5177
ACTN
WUPA
CG9471
CG7378
ROE1
EIF1
RPT5
RPT2
JAR
CYPE
CG8728
BLP
SERRS
CCT8
GLYRS
CCT2
ZASP52
ZASP66
CLIP-190
CG3270
PAR-1
TWS
CCT1
AK2
MTHFS
PRAT2
BAF
TYRRS
GART
ADSL
MBS
PP1-87B
WDB
CANB
KLC
HTS
LAM
HDAC1
CTBP
SAM-S
PKC98E
PKC53E
NCA
ENS
CASK
HIS2AV
MAP205
EMB
TRAL
CG30122
SYNJ
ME31B
YPS
GEL
BRP
RAB3
RCC1
SRP54
SM
NTF-2
SAP47
DRP1
TFAM
RAB10
YTR
SMD2
SRRM1
OPA1
CG32544
SNRPG
HFP
CG5808
