# Supplementary material for: Cryptochrome interaction networks across different tissues in Drosophila melanogaster
Source: Biol Direct. 2025 Nov 28;20:114. doi: 10.1186/s13062-025-00696-x (PMC12661795; doi:10.1186/s13062-025-00696-x)
Supplement: Supplementary file 5 — Supplementary Material 5 [file 13062_2025_696_MOESM5_ESM.pdf]

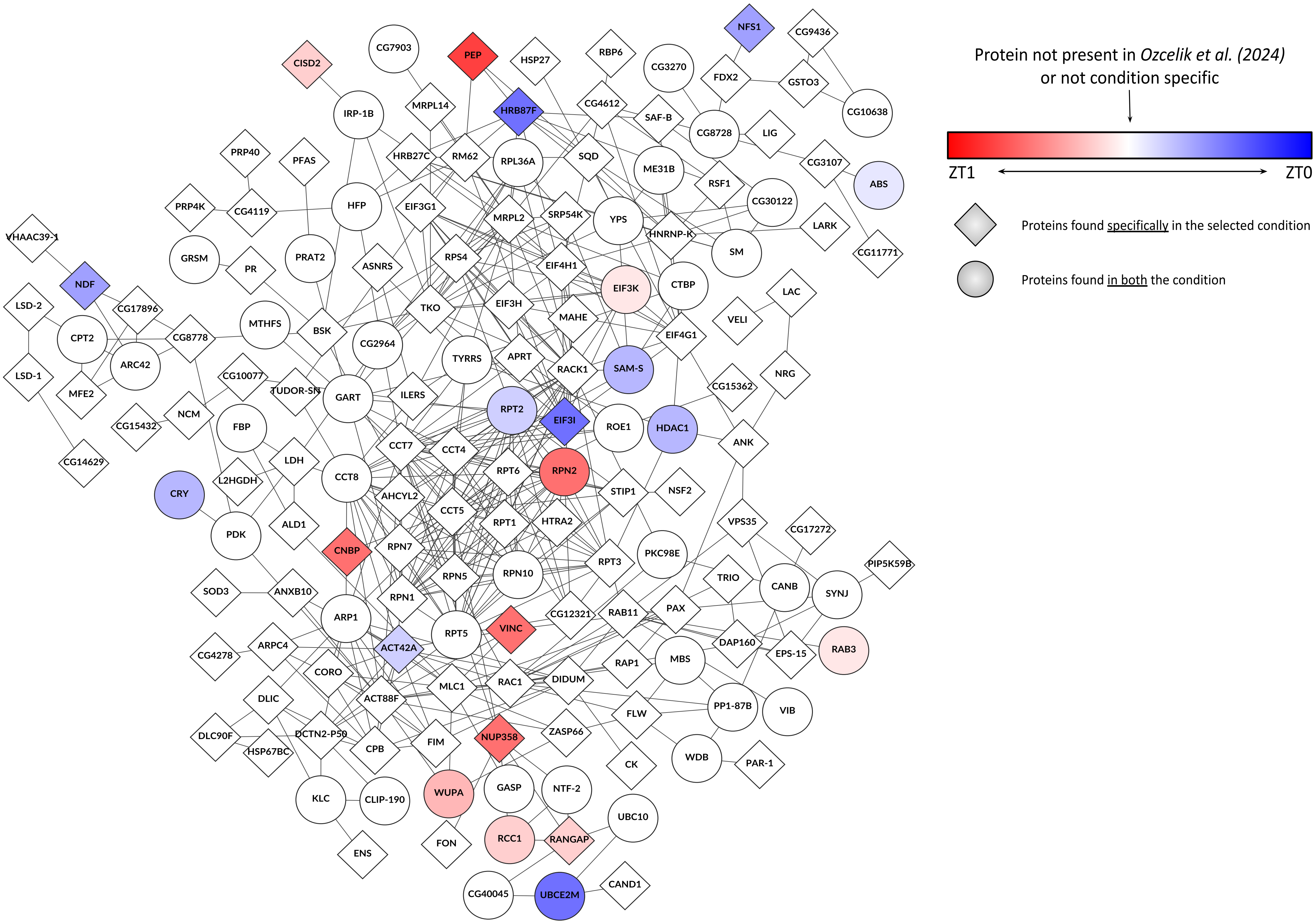

Protein not present in *Ozcelik et al. (2024)*
or not condition specific
ZT1
ZT0
Proteins found specifically in the selected condition
Proteins found in both the condition
NFS1
CG9436
CISD2
CG7903
PEP
HSP27
RBP6
CG3270
FDX2
GSTO3
CG10638
IRP-1B
MRPL14
HRB87F
CG4612
SAF-B
CG8728
LIG
CG3107
ABS
PRP40
PFAS
HRB27C
RM62
RPL36A
SQD
ME31B
RSF1
CG30122
PRP4K
CG4119
HFP
EIF3G1
MRPL2
SRP54K
YPS
HNRNP-K
SM
LARK
CG11771
VHAAC39-1
GRSM
PR
PRAT2
ASNRS
RPS4
EIF4H1
EIF3K
CTBP
NDF
LSD-2
CG17896
MTHFS
BSK
TKO
EIF3H
MAHE
VELI
LAC
CPT2
CG8778
CG2964
TYRRS
APRT
RACK1
SAM-S
EIF4G1
ARC42
CG10077
TUDOR-SN
GART
ILERS
RPT2
EIF3I
ROE1
HDAC1
CG15362
NRG
LSD-1
MFE2
CG15432
NCM
FBP
ANK
CCT7
CCT4
LDH
CCT8
RPT6
RPN2
STIP1
NSF2
CG14629
CRY
L2HGDH
AHCYL2
CCT5
RPT1
HTRA2
VPS35
CG17272
PDK
ALD1
CNBP
RPN7
PKC98E
RPT3
TRIO
CANB
SYNJ
PIP5K59B
RPN5
RPN10
SOD3
ANXB10
RPN1
ARP1
CG12321
RAB11
PAX
RPT5
VINC
DAP160
EPS-15
RAB3
CG4278
ARPC4
ACT42A
CORO
MBS
RAP1
MLC1
RAC1
DIDUM
DLIC
ACT88F
PP1-87B
VIB
FLW
DLC90F
DCTN2-P50
HSP67BC
CPB
FIM
NUP358
ZASP66
CK
WDB
PAR-1
KLC
CLIP-190
WUPA
GASP
NTF-2
UBC10
ENS
FON
RCC1
RANGAP
CG40045
UBCE2M
CAND1
